# Genomic population structure and prevalence of copy number variations in South African Nguni cattle

**DOI:** 10.1186/s12864-015-2122-z

**Published:** 2015-11-04

**Authors:** Magretha Diane Wang, Kennedy Dzama, Charles A. Hefer, Farai C. Muchadeyi

**Affiliations:** Department of Animal Sciences, University of Stellenbosch, Private Bag X1, Matieland, Stellenbosch, 7602 South Africa; Biotechnology Platform, Agricultural Research Council, Private Bag X5, Onderstepoort, 0110 South Africa

**Keywords:** Breed diversity, Nguni cattle, Genetic variation, Adaptation

## Abstract

**Background:**

Copy number variations (CNVs) are modifications in DNA structure comprising of deletions, duplications, insertions and complex multi-site variants. Although CNVs are proven to be involved in a variety of phenotypic discrepancies, the full extent and consequence of CNVs is yet to be understood. To date, no such genomic characterization has been performed in indigenous South African Nguni cattle. Nguni cattle are recognized for their ability to sustain harsh environmental conditions while exhibiting enhanced resistance to disease and parasites and are thought to comprise of up to nine different ecotypes.

**Methods:**

Illumina BovineSNP50 Beadchip data was utilized to investigate genomic population structure and the prevalence of CNVs in 492 South African Nguni cattle. PLINK, ADMIXTURE, R, gPLINK and Haploview software was utilized for quality control, population structure and haplotype block determination. PennCNV hidden Markov model identified CNVs and genes contained within and 10 Mb downstream from reported CNVs. PANTHER and Ensembl databases were subsequently utilized for gene annotation analyses.

**Results:**

Population structure analyses on Nguni cattle revealed 5 sub-populations with a possible sub-structure evident at K equal to 8. Four hundred and thirty three CNVs that formed 334 CNVRs ranging from 30 kb to 1 Mb in size are reported. Only 231 of the 492 animals demonstrated CNVRs. Two hundred and eighty nine genes were observed within CNVRs identified. Of these 149, 28, 44, 2 and 14 genes were unique to sub-populations A, B, C, D and E respectively. Gene ontology analyses demonstrated a number of pathways to be represented by respective genes, including immune response, response to abiotic stress and biological regulation processess.

**Conclusions:**

CNVs may explain part of the phenotypic diversity and the enhanced adaptation evident in Nguni cattle. Genes involved in a number of cellular components, biological processes and molecular functions are reported within CNVRs identified. The significance of such CNVRs and the possible effect thereof needs to be ascertained and may hold interesting insight into the functional and adaptive consequence of CNVs in cattle.

**Electronic supplementary material:**

The online version of this article (doi:10.1186/s12864-015-2122-z) contains supplementary material, which is available to authorized users.

## Background

Copy number variants (CNVs) are segments of DNA that are 1 kb or larger in size and display a variable copy number relative to a reference genome, hence comprising deletions, duplications and insertions [1]. A number of recent studies demonstrated CNVs to be prevalent in bovine genomes [2, 3]. CNVs are reported to affect a greater percentage of genomic sequences and have been identified in regions covering a number of genes that are recognized to play a role in cattle environmental responses and adaptation [4]. CNV region (CNVR) incidence also demonstrates some tendancy to parallel breed history and breed formation patterns [4, 5].

The development and focus on intense selection programs have greatly enhanced the genetic improvement of a number of domesticated cattle breeds worldwide. Understanding the multiple components of functional breed diverstiy have important implications for breed management and genetic improvement practices, especially in breeds that are locally adapted and have not undergone intense artificial selection. South African Nguni cattle represent such a distinct, conserved, Sanga type cattle breed that has undergone little synthetic breeding [6, 7]. Having endured natural selection pressures from a variety of disease agents and harsh climatic conditions, Nguni cattle have proven to prevail in suboptimal environmental circumstances [8]. These indigenous South African cattle are also recognized for their small frame size and diversely patterned and multi-coloured hides.

The availability of two cattle reference genomes (Btau_4.0 and UMD3.0) (The Bovine Genome sequencing and analysis consortium, [9]) and the development of genomewide single nucleotide polymorphism (SNP) genotyping arrays has enabled new avenues of research in bovine genomics. Although SNPs have been the primary focus of variant screening and association analyses, the recent development of CNV discovery tools utilizising both sequencing and SNP data hold opportunity for the in depth investigation into the prevalence of additional types of genomic variation [10–12].

The role that CNVs play within breeds to ensure diversity and adaptation has not yet been investigated. Nguni cattle have undergone scant synthetic breeding and are well adapted to their primary environment. With CNVs demonstrating a possible correspondence with breed diversity and adaptation, Nguni cattle present a valuable breed in which to investigate CNV prevalence and distribution.

This study investigated the population structure, haplotype block structure and the occurance and distribution of CNVs in Nguni cattle of South Africa using genotype data from the Ilumina Bovine SNP50K panel. Extensive linkage disequilibrium studies have been performed in cattle [13, 14]. Haplotype block (HPB) structure studies are however not as widespread [15]. The characterization of HPB structures at the population level contribute towards understanding the nature of non-linear association between phenotypes and genes [15]. This study determined the prevalence of CNVs within Nguni cattle followed by an analysis of their distribution within the different ecotypes inferred by population structure analysis. The prevalence of HPB structures in CNV formation was also investigated.

## Results and discussion

### SNP quality control

The Illumina Bovine SNP50 beadchip v2 comprising of 54,609 markers was utilized in the study (Illumina Inc., San Diego, CA). Of these 54,609, 54,060 SNP probes map to the most current UMD 3.0 bovine reference genome. After genotyping, a total of 1,340 variants were removed due to missing genotypes, and a further 11,232 variants were removed due to having a minor allele frequency of less than 0.02 and an additional 1,724 variants with a call rate of less than 95 % in the sampled population. In summary, 40,313 SNPs remained after applying extensive quality control (QC) pruning.

### Population structure analysis

#### Population structure QC

**Fig. 1:**
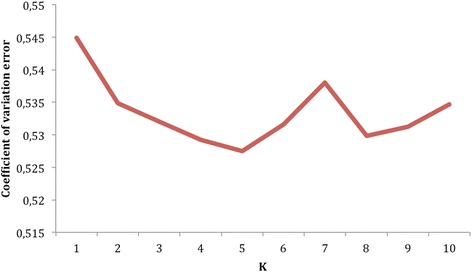
Cross-validation plot demonstrating the effect of different K-values on the cross-validation error

The 40,313 SNPs that remained after QC were further pruned for linkage disequilibrium (LD) using a threshold of *r*^*2*^ = 0.1. LD trimming resulted in another 29,836 SNPs pruned from the dataset, resulting in a final set of 10,477 SNPs used in the downstream analysis. Of the 492 animals sampled, 230 demonstrated an identy by descend (IBD) value of greater than 0.25 with animals within the dataset and were subsequently removed. Two hundred and sixty two unrelated animals thus remained for population structure analyses. Previous research suggests Nguni cattle populations to comprise of up to 9 different eco-types [6]. This estimation was then used to perform for a cross validation for 10 different K values. Standard error estimates for K ranged from 0.545 for K = 1 to 0.527 for K = 5 (Fig. 1).

#### Population structure statistics and classification

**Fig. 2:**
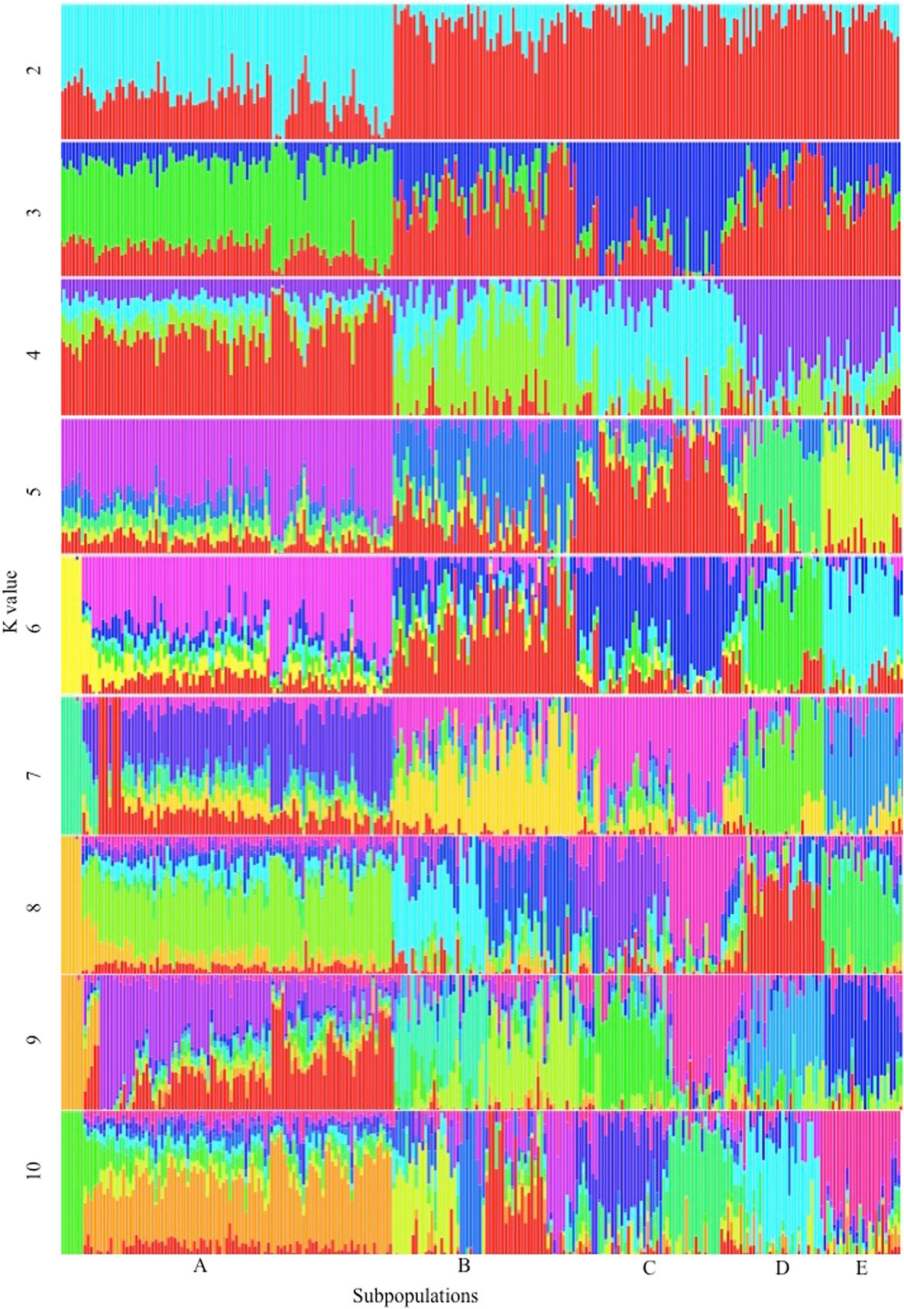
Boxplot demonstrating the population structure of the Nguni cattle for k = 2 to k =10

**Fig. 3:**
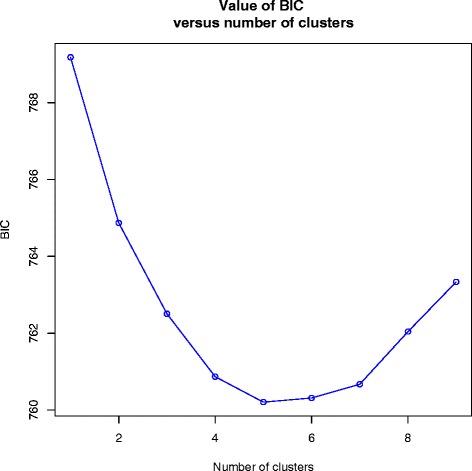
A linear graph demonstrating the bayesian information criterion against the number of clusters

**Fig. 4:**
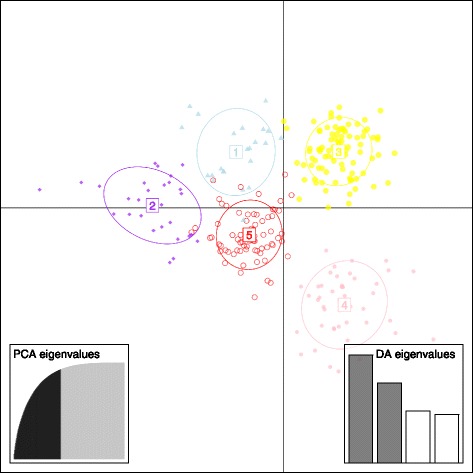
A DPCA plot demonstrating the group clustering with the subfigure 1 and 2 exhibiting discriminant eigenvalues and PCA eigenvalues

Organization of the data according to ancestry percentages, demonstrated 5 distinct sub-population clusters (Fig. 2). Instead of exhibiting the typical “v” shape graph which congests at the optimal K, the K graph demonstrated a “w” type of formation, with K equal to 8 (K8) following closely behind the optimal of K5. Admixture between sub-populations was evident. Sub-populations were assigned alphabetical tags. Nguni cattle have only recently been incorporated into synthetic breeding schemes, and for many years subsisted under natural selection pressures [16]. It can thus be expected that crossing between eco-types would be evident. The observed clustering may therefore be subsequent to such crossing between ecotypes or an indication of subpopulations that diverged more recently from one another. It is however, important to note that the ecotype structure of the studied animals was unknown upon sampling of animals used in the analyses. Discriminant Principle component analyses (DPCA) also demonstrated 5 clusters within the 262 Nguni animals and is presented in Figs. 3 and 4.

### Haploblock analysis

#### Haploblock statistics

A haplotype block is a combination of allelles that are linked on a common chromosome and inherited concurrently from a single ancestor [17]. Five hundred and fourty one haplotype blocks were identified across all 492 animals. Of these, 297 covered 3 or more SNPs. HPBs ranged in length from 84 base pairs on chromosome 8 to 199,730 base pairs on chromosome 1 (Table 1). The average length of the haplotype blocks was 79,686.68 (SD ± 67,651.42) base pairs across chromosomes with a total HPB length of 41.5Mbp. Large amounts of variation in haplotype structure and size between chromosomes were observed. Chromosome 1, 2, 3 and 8 exhibited the most haplotype blocks at 43, 33, 37 and 30 respectively (Table 1). Althought the largest HPB was found on chromosome 1, chromosome 10 contained the highest average HPB length of 123 kbps and the second highest percentage of its genome comprising of HPBs (Table 1). Previously, a negative correlation was reported between the average HPB length and recombination rate [18], and there also exists evidence of differences in recombination rates between cattle breeds [19].

**Table 1:** Haplotype block chromosomal distribution and characteristics

CHR	CHRLN	SNP	HPB	MinL	MaxL	AvL	HPBLn	PCN
1	161428367	2637	43	2809	199730	10561715	4330346	2.68
2	141965563	1691	33	1641	190889	77656.88	2485053	1.75
3	126844711	1716	38	108	192734	84265.08	3117846	2.46
4	123809850	1358	22	3406	198460	99861.67	2097117	1.69
5	125249322	1349	21	148	191441	80559.43	1691769	1.35
6	122519025	1438	21	2660	197610	82146.24	1725092	1.41
7	113029157	1328	31	1969	199428	88447.73	2653463	2.35
8	116846264	1306	30	84	197993	95895.72	2781006	2.38
9	108503706	1253	23	449	173897	61200.77	1346440	1.24
10	105982576	1059	22	10923	194738	123553.18	2718192	2.56
11	109987751	1071	15	9816	191201	96847.67	1452730	1.32
12	85119472	2140	25	620	194123	75741.70	1817826	2.14
13	84213851	1220	16	382	196561	57314.19	917043	1.09
14	81216349	1121	25	108	160297	48024.25	1152607	1.42
15	84472747	1015	10	6788	176328	73205.13	585651	0.69
16	77710258	852	22	178	188386	74494.59	1638903	2.11
17	76280064	987	12	2032	194454	65094.33	781144	1.02
18	65811054	769	13	5603	193175	63212.5	758563	1.15
19	64845320	864	14	1014	197085	82993.29	1161920	1.79
20	75686341	756	12	1527	195895	72733.91	800085	1.06
21	69078422	755	10	12797	173099	86163.67	775483	1.12
22	61598339	819	9	9271	176207	49994	399961	0.65
23	52334015	1980	11	2414	142956	48732.73	536071	1.02
24	64508398	1950	12	95	195261	65562.08	786757	1.22
25	44081797	1650	11	1343	180067	56038.46	616434	1.40
26	51826547	2017	11	281	187010	85746.2	857473	1.65
27	48460478	1784	8	151	191285	70888.75	567118	1.17
28	45964680	1890	10	675	140455	41588.63	332719	0.72
29	51812796	1538	11	3683	172838	64737.73	712126	1.37
Tot		40313	541	84	199730	79686.58	41596938	

Chromosome number (CHR), chromosome length (CHRLN), number of SNPs (SNP) and HPBs (HPB), minimum length (MinL), maximum length (MaxL), average length (AvL) and total length (HPBLN) of HPBs and percentage of chromosome covered by HPBs (PCN)

The smallest number of haplotype blocks were identified on chromosomes 22, 27 and 28, with chromosome 22 exhibiting the smallest percentage of its length consisting of HPBs. The exact boundaries of HPBs are not resilient to variations in SNP density as the average size of HPBs may decrease with the greater sequence coverage of the HPB that results from elevated marker density [20]. Khatkar et al. [21] reported 727 haplotype blocks covering more than 3 SNPs in 1000 Holstein-Friesian bulls using 9195 SNPs in Hardy-Weinberg equilibrium mapped to the Btau 3.1 bovine assembly. Haploblocks reported in this study were on average 1 kb larger than those reported by Khatkar et al. [21].

#### Haploblock gene ontology

Haplotype blocks have discrete boundaries that are defined by recombination hotspots [22]. In the past HPB analyses were primarily used to identify tag SNPs [23]. In this study 232 genes were present within the 541 HPB identified (Additional file 1). Five genes, including Bos taurus fat mass and obesity associated (FTO), family with sequence similarity 155 (FAM155A), Glypican (GPC5), Na+/K+ transporting ATPase interacting 2 (NKAIN2), UDP-N-acetyl-alpha-D-galactosamine:polypeptide N-acetylgalactosaminyltransferase-like 6 (GALNTL6) and cysteine conjugate-beta lyase 2 (CCBL2) were covered by two separate haplotype blocks lying in close proximity to each other (Additional file 1). We used gene ontology (GO) terms to classify these genes into a number of biological process, molecular functions and cellular components. Furthermore, we used the PANTHER database to identify protein features associated with GO terms (Additional file 2). A total of 122 genes involved in metabolic processes and 143, 226 and 188 genes involved in biological regulation and biological process and cellular processes respectively were positioned within HPB regions ascertained. Of interest were genes involved in immune system process (18), immune response (7), immune system development (9) and positive regulation of response to stimulus (17) (Additional file 2). Gibson et al. [24], utilised exome-chip data to demonstrate patterns of linkage disequilibrium and subsequent haplotype structure to be informative of gene function and possible relationships between genes and specific phenotype clusters. Nguni cattle are suited to survive in harsh environmental conditions with enhanced disease and parasite resistance as well as heat tolerance [25]. It is therefore not surprising that genes involved in processes like immunity and stimulus responses lie within the HPBs identified.

### CNV identification

#### CNV model quality control

**Fig. 5:**
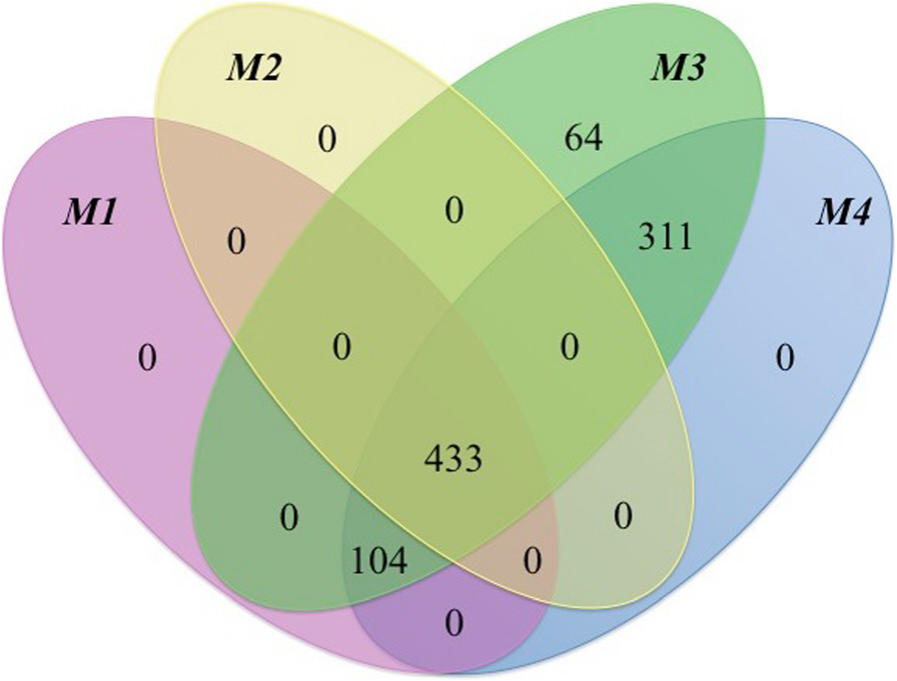
A venn diagram showing the overlap of CNVs identified by each of the four models in 492 Nguni cattle

As with all current CNV detection methodologies deducing copy number variations from SNP data encompasses a number of areas at which error can be introduced and ascertainment biases presented [26; 27]. The bovine SNP50 beadchip is limited to detected variations in the copy numbers of sequences present in the reference population that was used to design the probes, while it does not provide details regarding the location of duplicated copies [28]. A number of factors influence the accuracy of CNV breakpoint detection, including batch effects, population stratification, experimental differences and the robustness of the statistical model [29]. SNPs utilized were also selected to have a minimum minor allele frequency and tend to be those that segregrate within multiple breeds [30]. The tendency of SNP arrays to demonstrate greater sensitivity to deletions than duplications is particularly note worthy in areas with insufficient probe density to use B allele frequency measurements which may result in the majority of the smaller CNV events being deletion events partially owing to an ascertainment bias [28]. With this in mind, four models utilizing different filtering stringencies were used to identify CNVs in Nguni cattle (see Methods) and are presented in Table 2. Four hundred and thirty three CNVs were identified by all four filtering techniques in 231 animals (Table 2). Discrepancies in the number of CNVs identified by each of the models was evident. Model 1 identified 353 CNVRs in the 379 animals that had a average length of 259 kb (Table 2). Inclusion of the gcmodel enabled additional animals to pass QC filtering and subsequently corresponded with an elevated number of CNVs being identified. Great variation in the size and number of CNVRs has been reported in cattle [31, 32]. CNVs in this study ranged from 30 kb to 1 Mb in size (Table 3). All models demonstrated a similar pattern of CNV numbers across animals, although models 3 and 4 determined a number of novel CNVs. All CNVs identified by models 1 and 2 were identified by either model 3 or 4 or by both (Fig. 5).

**Table 2:** Summary statistics of four CNV detection filtering models

MDL	GCWF	DLRS	GCMDL	ANMLs	QCPS	ANMLsCNVs	CNVRs	AvL
1	0.040	0.300	Yes	492	379	281	353	259283.62
2	0.040	0.300	No	492	326	231	334	270939.14
3	0.070	0.318	Yes	492	453	361	501	237869.23
4	0.070	0.318	No	492	462	352	486	240572.18

The stringencys (GCWF and DLRS), the number of animals (ANMLs), the number of animals that passed (QCPS), the number of animals with CNVs present in their genome (ANMLsCNVs), the number of CNVRs and the average length (AvL) of the CNVs identified within Nguni cattle

**Table 3:** Chromosomal distribution of CNVs identified in 492 Nguni cattle

CHR	CNVRs	CNVLN	PERCN	MinL	MaxL	AvL
1	34	4533994	2.81	36419	680994	133352.76
2	16	1884357	1.33	44214	260334	117772.31
3	19	4020748	3.17	53857	949810	211618.32
4	23	3218422	2.60	48441	397435	139931.39
5	15	1655058	1.32	47847	257875	110337.20
6	25	4303075	3.51	31128	953806	172123.00
7	11	1792440	1.59	52476	306135	162949.09
8	6	794463	0.68	76217	237689	132410.50
9	11	1230570	1.13	30336	289059	111870.00
10	7	822052	0.78	44415	184185	117436.00
11	13	1265163	1.15	52654	199903	97320.23
12	19	2775332	3.26	48596	392714	146070.11
13	6	1295356	1.54	86589	522669	215892.67
14	12	2133059	2.63	48512	741197	177754.92
15	11	1539814	1.82	51632	390973	139983.09
16	10	1379434	1.78	40032	242142	137943.40
17	10	2570441	3.37	74327	1285287	257044.10
18	3	298969	0.45	63682	161641	99656.33
19	3	415596	0.64	106928	182010	138532.00
20	11	1615406	2.13	49902	378113	146855.09
21	9	964270	1.40	42434	156070	107141.11
22	6	1942282	3.15	73778	1171794	323713.67
23	4	506937	0.97	42345	211284	126734.25
24	12	1744861	2.70	38738	343135	145405.08
25	4	1369746	3.11	66262	1041448	342436.50
26	11	1958085	3.78	73168	518655	178007.73
27	7	784830	1.62	50958	261955	112118.57
28	7	1354237	2.95	117087	414660	193462.43
29	9	1179028	2.28	54840	367944	131003.11
Total	334	51348025		30336	1285287	153736.60

CNVR count (CNVRs), total length (CNV,LN), percentage of chromosome length (PERCN) and minimum (MinL) maximum (MaxL) and average (AvL) lengths of CNVRs identified on each of the 29 Btau chromosomes of 492 Nguni cattle

#### CNV statistics

Only those CNVs identified by all models were utilized for further analyses, to ensure validity of variable regions. Only 326 animals passed the PennCNV filtering. A total of 334 CNVRs were identified across models in 231 of these animals (Table 2). CNVR were between 30 kb and 1.2 Mb in length (Table 3). We identified 90 animals that contained a single copy number variation in their entire genome. One animal contained 22 CNVs in its genome. The average number of CNVs per animals was 2.61 (SD ± 2.63) which is similar to the 3.2 CNVs per animal reported by Bae et al. [34] in Korean cattle. Those animals with multiple CNVs detected in their genome, demonstrated a seemingly random spread of CNVs across chromosomes. Overall, 334 CNVRs were identified in 231 animals which was notably less than the 281 and 3088 CNVs identified by Hou et al. [5] and Hou et al. [31] respectively in 39 and 47 animals from a variety of African breeds. The smallest CNV was 30kbs in length and demonstrated a single copy duplication (Table 4). Single copy deletions were identified in most of the animals while only 1 animal had a double copy duplication. This discrepancy in copy number of CNV may be an artifact of the PennCNV algorithm which has been seen to identify many more deletions than duplications [33]. SNP array platforms tend to also demonstrate reduced precision in detecting single copy gains relative to deletions, of which this may be an artifact [28]. Jiang et al. [32] identified 367 CNVRs comprising of 232 deletions, 111 duplications and 15 CNVRs of both gain and loss events by means of PennCNV analyses of high-density SNP genotyping data from 96 Chinese Holsteins. Hou et al. [5] on the other hand, reported 682 CNVRs encompassing 370 loss, 216 gain and 96 loss and gain events in the same region in 521 animals representing 21 different breeds, also based on SNP genotyping arrays. Although Jiang et al. [32] highlighted the differences in size and structure of populations, a difference in platforms and algorithms used and CNV discovery and filtering techniques also contributed to such incongruities. When CNVs from this study were compared to CNVs published in four other studies, very little overlap in the exact CNV breakpoints existed between studies. A number of CNVs identified in this study were however positioned in close proximity (<1 Mb) to those CNVs identified by Bae et al. [34], Bickhart et al. [35], Fadista et al. [36] and Hou et al. [5] in other cattle breeds. This clustering of CNV regions demonstrated the potential for certain regions of the genome to be more susceptible to copy number variations within cattle breeds. The form and exact locality of these CNVs may be what contributes to the nature and degree of variation exhibited by gene expression of adjacent genes. Fadista et al. [36] reported CNV distribution in cattle to reflect chromosomal size with the most CNVs being identified on the largest chromosomes. Our data, however does not follow this pattern entirely. Chromosome 6 had the greatest number (18) of CNVs while chromosome 18 contained no CNVs (Table 3). This reflects findings of Guryev et al. [37], who reported chromosome 18 to be a “cold spot for CNVs” in rats. Chromosome 18 together with chromosomes 5, 27 and 29 are reported to demonstrate a preponderance of segmental duplications in the bovine genome [2]. A noticeable feature of CNVs, particularly larger CNVs, is their prevalence in regions with known segmental duplications [35]. Also known as low copy repeats (LCRs), these segmental duplications are duplicated fragments of DNA that are more than 1 kb in size and can be found either on the homologous chromosome or on a separate, non-homologous chromosome with a minimum of 90 % sequence identity [38]. In this study we identified 11, 0, 6 and 4 CNVRs on chromosomes 5, 18, 27 and 29 respectively. SNPs were reported as being sparse in regions of segmental duplications and may explain the comparatively lower numbers of CNVs on these chromosomes [39]. Segmentally duplicated domains are known to encode protein products that play a prominent role in species adaptation [40], which makes identification of CNVs in these regions crucial. Techniques such as next generation sequencing may be more suitable for the detection of CNVs, particularly on chromosomes previously reported to harbour low number of CNVs.

**Table 4:** Summary statistics of CNV deletions and duplications

CN^a^	ANMLs	CNVs	MinL	MaxL	AvL
0	16	7	44415	76444	53931.94
1	406	308	36419	1053438	143300.88
3	179	142	30336	953806	164468.69
4	1	1	102466	102466	102466

The number (CNVs), minimum length (MinL), maximum length (MaxL) and average length (AvL) of CNVs

^a^double deletion (CN = 0), single deletion (CN = 1), single duplication (CN = 3) and double duplication (CN = 4)

#### Gene ontology

Four hundred and fifty eight genes located within 10 Mb of CNVRs were identified. A number of genes including Milk fat globule-EGF factor 8 protein (MFGE8), collagen type XIII alpha 1 (COL13A1), cystic fibrosis transmembrane conductance regulator (CFTR), Bradykinin receptor B1 (BDKRB1), prostaglandin-endoperoxide synthase 2 (PTGS2), major histocompatibility complex, class I-related (MR1), Platelet/endothelial cell adhesion molecule 1 (PECAM1) and leucine rich repeat and fibronectin type III domain containing 5 (LRFN5) involved in immune system response or B-cell mediated immunity were overrepresented within identified CNVs (Additional file 3). Copy number variations in immune related genes have previously been linked to disease [36]. Variation in the genes comprising the major histocompatibility complex have been reported to play a pivotal role in the predisposition of cattle to diseases such as dermatophilosis, mastitis and tick born infections [41]. Stothard et al., [42] reported CNVs that are closely associated with immune and lactation genes. Bickhart et al. [35] reported that 15 of the 25 most variable copy number genes they identified, had functions associated with immune response and host defense, such as defensin, interferon and GIMAP (GTPase and IMAP) families. Anhidrotic ectodermal dysplasia in cattle is associated with a deletion that may range between 2 and 160 kb of the genome and includes third exon of the EDA gene [43]. Flisikowski et al. [44] demonstrated a 110 kb microdeletions in the MER1 repeat containing imprinted transcript 1 ( MIMT1) gene region to be linked to the incidence of abortions and stillbirths in cattle. A 2.8 kb deletion in the solute carrier family 4 (anion exchanger), member 2 ( SLC4A2) gene was reported by [45] to cause osteopetrosis in Red Angus cattle. Two causal deletions in the claudin 16 ( CLDN-16) gene were linked to renal tubular dysplasia in Japanese black cattle [46].

Sixteen CNVRs were detected in 8 or more animals in this study (Table 5 and Additional file 4). These CNVRs contained a number of genes involved in immune system processes, cell communication, response to toxic substances and cell communication. The CNVR on chromosome 1 located between base pair 104,798,012 and 105,264,358 observed in multiple animals contained the sucarse-isomaltase (SI), intestine-specific gene (Additional file 4). Nguni cattle are reported to exhibit a superior feed conversion rate when compared to other indigenous breeds [47].

**Table 5:** Copy numbers and gene names of CNVRs present in 5 or more individuals

CNVR	CN^a^				Total	GEN
	0	1	3	4		
chr17:73713062-74998349		8	13		21	CHCHD10 IGLL1 LOC527441 SLC5A1 VPREB3 ZNF280A ZNF280B ZNF70 DERL3 GSTT1 GSTT3 GSTT4 MIF SLC2A11 SMARCB1 DDT GGT1 GGT5 SUSD2 C17H22orf13 LOC531152 MIR2323 RTDR1 SNRPD3 SPECC1L UPB1
chr1:104798012-105264358		16	1		17	SI
chr24:28154039-28497174		13	3		16	CDH2
chr7:75305297-75370366	1	8	5		14	GABRG2
chr5:3260057-3434356		7	4	2	13	ATXN7L3B
chr6:43037439-43089739	12	1			13	GBA3
chr19:49657396-49784054		10	2		12	LYAR NSG1 OTOP1 STX18 TMEM128 WDR1 ZBTB49
chr6:108998175-109951981		5	7		12	NOT_FOUND
chr9:3651455-4439872		10	1	1	12	PECAM1 POLG2
chr1:32509969-32781614	1	7	3		11	
chr6:71910076-72118486			11		11	CHIC2
chr28:21101833-21762976		5	5		10	CTNNA3
chr22:59487979-60960603		8	1		9	
chr6:53514737-53692295		9			9	ACAD9 C22H3orf37 CNBP COPG1 EFCC1 GATA2 ISY1 MIR2374 RAB7A RPN1 EFCC1 IQSEC1 ISY1 CHCHD4 HDAC11 NUP210 TMEM43 WNT7A XPC
chr14:54875898-55141942			8		8	ANGPT1
chr25:41191025-42687812		5	3		8	BRAT1 CARD11 GNA12 GRIFIN LFNG MIR2390 MIR2890

^a^ double deletion (CN = 0), single deletion (CN = 1), single duplication (CN = 3) and double duplication (CN = 4)

CNVs have potential to not only change gene dosage and structure, but may modify gene regulation as well as expose recessive alleles [48]. A total of 458 genes were located adjacent to (within 10 Mb), or within an identified CNV. Comparison of those genes contained within CNVRs identified within this study with those identified within other breeds [5, 29, 30] revealed 402 (87 %) genes that were unique to the Nguni (Additional file 5). The only gene identified close to a CNVR in all four studies was immunoglobulin lambda-like polypeptide 1 (IGLL1). IGLL1 is one of the polypeptides of the immunoglobulin light chain gene pool in domestic cattle that play a role in B cell production [49]. This gene lies adjacent to its associated colute carrier (SLC) polypeptide [49]. Immunoglobulins are the molecular mediators of the adapative humoral response of jawed vertebrates (Gnathostomata). The evident variation in copy number at this gene in a number of bovine breeds may explain the variation in the adaptive immunity evident between breeds, but further investigations into the role of this CNV needs to be ascertained. The *Bos taurus* pregnancy-associated glycoprotein (MGC157405) gene is the only gene represented across CNVRs of Hou et al. [5], Bickhart et al. [35] and this study and forms part of the cellular defense response. Ten genes are shared between this study and that of Hou et al. [5] and Bae et al. [34], including O-fucosylpeptide 3-beta-N-acetylglucosaminyltransferase (LFNG) and ADP-ribosylation factor-like 6 (ARL6) that are both involved in metabolic and cellular processes. B cell mediated immunity, mesoderm development and cell communication pathways also demonstrate representation by genes shared (Additional file 3). Twenty nine genes located within the Nguni CNVRs were also reported to be associated with CNVRs in Korean cattle [34] (Additional file 5). Overlapping genes were associated with a number of biological processes including positive regulation of cell proliferation, cell communication, detection of stimulus, cellular process, metabolic process and susceptibility to natural killer cell mediated cytotoxicity (Additional files 3 and 5). Thirteen of the genes associated with CNVRs in this study overlap with genes covered by CNVRs reported by Hou et al. [5] in a variety of cattle breeds, including African Breeds. The funtional annotation of these 13 genes were associated with immune system processes, cell communication and lipid metabolic processes (Additional files 3 and 5).

Five of the genes identified within CNVs in this study were also identified by Bae et al. [34] in 265 Korean cattle (Additional file 5) while another 5 corresponded to findings of Hou et al. [5] in multiple different Indicine, Taurine, Composite and African breeds. Bickhart et al. [35] speculated that the distinctions in selected breeds for specific traits could be linked to specific CNVs and that discrepancies in CNVs and subsequent CNVRs between different breeds could thus be expected. The greatest amount of gene overlap was between this study and that by Hou et al. [5]. This corresponds with the proposition of CNVs segregating within breeds as they analysed the greatest variety of cattle breeds (366 Taurine, 46 Composite, 70 Indicine and 39 African cattle) within their study.

Additional file 6 demonstrate biological process, cellular component and molecular functions that were represented by genes covered within CNVRs or lying within close proximity of CNVRs identified by all four models. The biological pathways with the greatest number of genes represented included biological process, primary metabolic process, cellular metabolic process, primary to stimulus and cellular process. Nervous system development (*p* = 0.008), single-organism behaviour biological pathways (*p* = 0.003) and dendrite cellular component (*p* = 0.05) demonstrated significant (*p* ≤ 0.05) overrepresentation. Genes involved in these processors were evident in CNVRs identified in all ecotypes. Hansen [50] denoted metabolic regulatory ability that results in a reduction in body temperature to be one of the factors that contribute to superior thermotolerance within cattle species. Whether the presence of CNVs at these genes may relate to the enhanced ability of Nguni cattle to handle harsh environmental conditions needs further investigation. Non-significant overrepresentation by CNV genes in 3055 biological processes, 593 molecular functions and 391 cellular components was evident. These systems included cellular response to transforming growth factor beta stimulus, regulation of B cell proliferation and  positive regulation of viral release from host cell functions.

Previous findings have demonstrated CNVRs to be located in areas containing genes associated with environmental responses like sensory, defense and immunological functions and regulatory processors [31, 51]. Similar patterns are evident within Nguni cattle and suggest CNVs to potentially play an important role in the adaptative traits evident in Nguni cattle populations.

#### CNVs and population structure

CNV characteristics for each subpopulation are presented in Table 6. Sub-population A had the highest average number of CNVs per animal while sub-population D had the smallest average CNV length. Sub-population A had the greatest number of animals in the study (*n* = 103) and also presented with the most CNVRs (*n* = 121) (Table 6). A number of CNVRs were shared between populations. The most widespread CNVR was identified on chromosome 6, covering the protocadherin 7 (PCDH7) and cysteine-rich hydrophobic domain 2 (CHIC2) genes and present in sub-populations A, B, C and E (Table 7). Increasing evidence has suggested that CNVs play a primary role in inter-individual diversity [52], attributing to both normal phenotypic variation and major variations in complex traits such as susceptibility to disease [53, 54]. Within Nguni cattle sub-populations a broad array of phenotypes are evident with great variations in coat colour, behaviour and immune response being evident [6]. As little research into the genotypic makeup of the Nguni ecotypes has been performed, little is known about what differentiates these ecotypes on a genomic scale. Eighteen CNVRs were identified in multiple animals and are reported in Table 5. On closer inspection of these CNVRs, some noteworthy association can be seen. The CNVR located on chromosome 1 (chr1:104798012–105264358) was identified in 7 animals. Four of the animals belong to sub-population A while 10 of the 11 animal genomes containing the CNVR on chromosome 4 (chr4:108834886–109130345) belonged to sub-population A. CNVR chr6:71910076–72118486 was present in 13 animals with 6 and 5 animals from sub-populations A and C respectively.

**Table 6:** Summary statistics of CNVs identified in five Nguni cattle subpopulations

Pop	ANMLS	ANMLsCNVs	CNVRs	Av/An	MinL	MaxL	AvL	No. Gen.
A	103	62	121	1.71	42164	1066850	171789.26	39
B	57	27	39	0.98	62327	741252	186667.09	5
C	53	26	39	1.26	50170	518655	167637.18	65
D	23	6	8	0.39	82202	180684	146892.13	50
E	25	12	20	1.44	42164	1066850	223319.41	195
Total	261	133	268	1.32	42164	1066850	178994.23	339

The number of animals (ANMLs), animals with CNVs (ANMLsCNVs), CNVRs (CNVRs), the average number of CNVRs per animal (Av/An) the minimum (MinL), average (AvL) and maximum (MaxL) lengths of CNVs and the number of genes (No. Gen)

**Table 7:** CNVR genes of Nguni cattle subpopulations

Pop	No. Gen.	GEN
A B C E	2	PCDH7 CHIC2
B C E	1	ATXN7L3B
A C E	1	NXNL2
A B C	4	TNFAIP8 CTNNA3 SI LOC780933
C E	2	KCND3 ATP5G3
B E	1	ARL6
A E	17	RAB40C KLHL1 CISD1 IPMK PWWP2B MRPL28 VPREB3 DECR2 TRNT1 PCDH10 ARL4C ZNF70 NME4 CHCHD10 IGLL1 TMEM8A OTOP1
A D	2	CLRN1 LRFN5
B C	2	HPS3 LOC514194
A C	8	BICD2 CENPP ATG2B CDH12 BDKRB1 BDKRB2 ZWINT MR1
A B	11	GABRG2 PDLIM1 LOC509513 DCTD NDST4 CDH2 C28H10orf35 COL13A1 PROM1 ADCY1 TMPRSS15
E	14	GRAP2 SERPINB8 CADPS2 HERC4 ENTHD1 KCND2 PPP1R14C FKBP5 MSX1 CTSD FARS2 HTATSF1 NUP210 SORBS2
D	2	ASPH FSTL5
C	44	NUP35 URB2 HCK INSL6 PDPN PLGRKT PECAM1 ZC3H7B GDA MMS22L C6H4orf32 RHAG CPS1 TM9SF4 POFUT1 GLYATL3 SERINC1 GBE1 TM4SF18 IL1R2 C23H6orf141 CYB5R1 WBSCR17 CDH10 PHYHIPL ATF2 CNTNAP3 ADCY8 ANKRD50 CRISP2 FAM204A MRPS31 CD274 SPAM1 CELF4 KCMF1 CRISP3 HMGXB4 CDC73 KIF3B CELF2 RAB21 LACTB2 RANGAP1
B	28	KATNBL1 MPPED2 C15H11orf70 FAM5C SH3BP4 HLTF C21H14orf49 TYW3 PAQR3 CHRM5 MIR1256 GJA1 RPL37A GPC5 CLN5 UBE2U OXR1 FAM98A COX7C SMAD4 ACSL1 LPHN2 TNNI3K CRYZ EMC7 PET112 DHX29 CADM1
A	149	TBC1D19 PTGER3 SEC62 LOC527441 NR3C2 CA8 PFKP DDT STUB1 GGT1 AMPH FBXL16 WDR24 C15H11orf96 PRKAR2B TMEM128 RPUSD1 FAF1 NPRL3 LARGE GRB10 AXIN1 LUC7L C11H2orf28 PDIA2 PROP1 MSLN PLEKHA3 NOL4 PDGFD LYAR SPECC1L RNF185 AMY2B SUSD2 QRFPR POLR3K RFC3 ARL4A ACSL6 WFIKKN1 CLN8 ACYP2 SLC22A18 GBA3 MIR2390 FUBP3 SLC5A1 SNRPD3 C25H16orf13 SELM FGGY OTX2 KCTD16 PTGS2 CARD11 C1QTNF7 ARHGDIG DDI1 HAGHL MIF NAP1L4 MTRR H2AFY2 ALX1 ERICH1 CHTF18 FGF9 WDR1 PLEKHA1 GNG13 SRSF6 RRAGC ADIG SEMA3A UPB1 FZD1 SORCS3 NARFL LUZP2 SMARCB1 C15H11orf58 HBA SELPLG BCHE ZNF703 TMEM119 HBQ1 RGS11 MGAT4C LIN7C ITFG3 LMF1 OSTN TMEM225 GSTT4 ASS1 NRG3 ALKBH3 STAB2 CTXN3 RHBDF1 PATZ1 C21H14orf2 SNRNP25 INO80D PRR5L DRG1 ZBTB49 C17H22orf13 SLC25A21 METRN FAM173A ZNF280A KCNJ3 RHOT2 ST6GAL2 PPAP2B INPP5J GSTT3 GSTT1 QTRTD1 GGT5 HTRA1 CARS SEMA3C LOC615200 SOX2 CFTR ZNF280B PHLDA2 LPHN3 LYPLAL1 HBM LSAMP NXPH2 KCNQ1 LIMK2 SLC2A11 FAM195A GRIN3A CDKN1C DRD1 AGPAT9 PIK3IP1 DERL3 SMTN LOC516108 XRCC2

The number of genes (No. Gen), gene names (GEN) and the subpopulation (Pop)

Two hundred and eighty eight genes were identified to be associated with CNVRs in sub-populations A, B, C, D and E (Table 7). A number of genes only identified within specific sub-populations were present (Table 7). Sub-population A has the most (149) unique genes that are not recorded in the other sub-population groups. The ataxin 7-like 3B (ATXN7L3B) and tumor necrosis factor and alpha-induced protein 8 (TNFAIP8) genes were present in CNVRs in sub-populations B, C and E and A, C and E respectively and play a role in the immune system process, and the response to stress.

#### CNVs and haplotype blocks

**Fig. 6:**
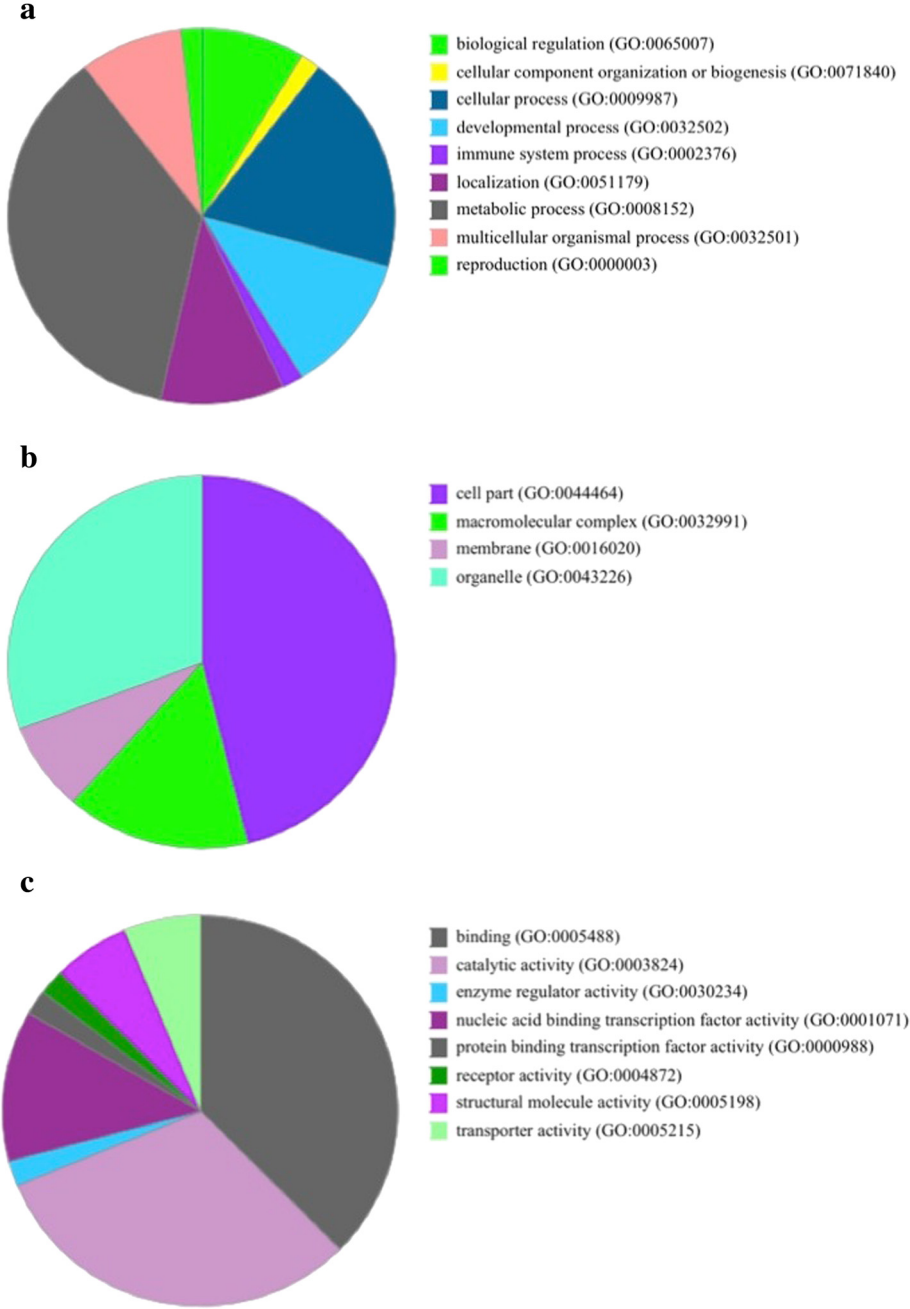
Panther pie chart of the (**a**) biological pathways, (**b**) cellular components and (**c**) molecular functions represented within genes of CNVRs that overlap or share breakpoints with HPBs

Thirty four HPBs lay either within, across or adjacent to CNVRs identified within the Nguni cattle population (Additional file 7). Half of these occurances were at CNVR sites that were present in multiple individuals, with one such CNVR on chromosome 1 that was present in 17 animals (Additional file 7). Another HPB overlaped a CNVR associated with genes Ly1 antibody reactive homolog (LYAR), neuron-specific protein family member 1 (NSG1), otopetrin 1 (OTOP1), syntaxin 18 (STX18), transmembrane protein 128 (TMEM128), WD repeat domain 1 (WDR1) and zinc finger and BTB domain containing 49 (ZBTB49) was present in 12 animals. Genes present in CNVRs that overlap or share cut-off points with HPBs contributed to a number of biological, cellular and molecular pathways (Fig. 6). Of the biological pathways, metabolic processes demonstrated the greatest gene representation. Other interesting biological pathways represented by genes covered by both HPB and CNVR were the immune system processes, biological regulation and cellular processes. Four cellular component pathways demonstrated representation. Of the molecular pathways represented, protein binding transcription factor had the greatest number of genes denoted within CNVR-HPB overlap regions. Other molecular functions of interest included receptor activity, enzyme regulator activity and catalytic activity.

CNVs have been reported to be in LD with surrounding SNPs, demonstrating conserved long-range haplotypes [55]. Meiotic crossing over hotspots flanked by recombinationally inert DNA is thought to be a major contributing factor in the presence of haplotype block structures [56]. Whether the mechanisms involved in meiotic recombination crossing-over may play a role in the variations in copy number is something that could be looked into as the exact mechanisms of CNV formation is yet to be fully understood.

## Conclusions

Population structure analyses revealed the presence of 5 subpopulations with some degree of admixture occuring between groups. A total of 334 CNVRs were identified and characterized within the genome of 492 Nguni cattle. Different filtering techniques were modelled. The inclusion of the gcmodel with the higher waviness stringency proved to demonstrate the greatest repeatability with CNVs identifed across models.

Eighteen CNVRs were identified in multiple animals. Among these regions, segregation within as well as across sub-population groups was evident. Specific CNVRs may play a role in the variation exhibited among Nguni ecotypes. Some of these CNVRs may also be distinct to Nguni cattle, contributing towards some of the distinctive phenotypic traits for which they are recognized. Until the twentieth century, Nguni cattle were primarily exposed to natural selection pressures and subsequently exhibit enhanced adaptive traits together with broad phenotypic diversity. Genes within CNVs demonstrated overrepresentation in a number of biological, molecular and cellular pathways and may therefore be potential contributors to the phenotypic diversity evident in Nguni cattle populations.

## Methods

### Sample collection and data generation

**Fig. 7:**
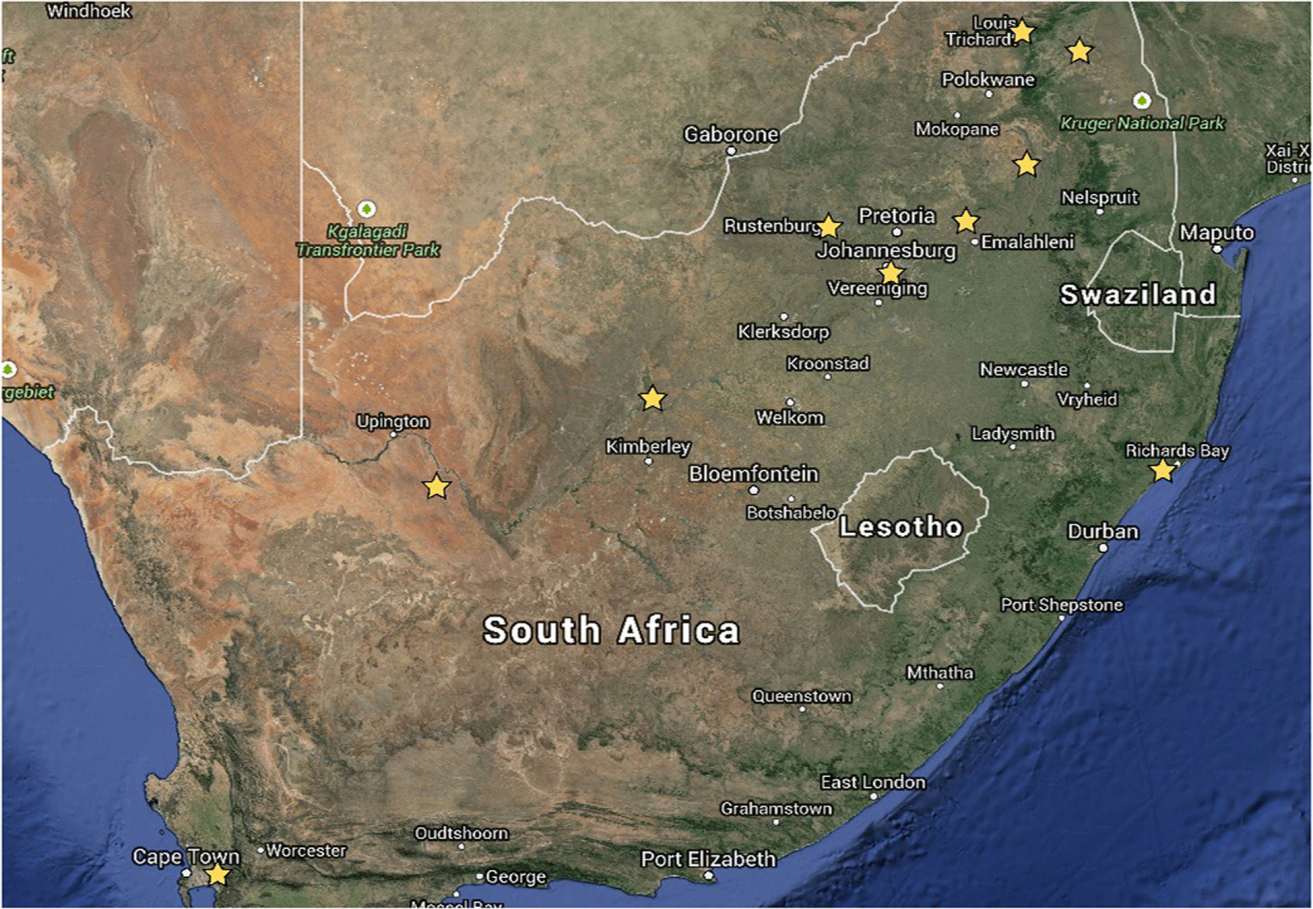
Geographic origin of the 492 Nguni cattle sampled in the current study

Blood samples were collected in 10 ml EDTA VACUETTE® tubes by means of venal puncture of the caudal vein from 492 Nguni animals distibuted across South Africa (Fig. 7). Genomic DNA was extracted by means of the Qiagen DNeasy Blood and Tissue Kit from the blood samples. The quantity and quality of extracted DNA was assessed by means of the Qubit and those samples exhibiting a minimum concentration of 50 μl were subsequently genotyped with the Illumina BovineSNP50 BeadChip (Illumina Inc., San Diego, CA) containing 54,001 highly informative markers that uniformly span the bovine genome. Illumina BovineSNP50 BeadChip SNP markers were designed based on the Btau 4.0 reference genome. Markers were clustered and genotyped by means of Illumina GenomeStudio v2.0 software. Fifty four of the genotyped samples were derived from a previous study [7] and were approved for this research by the University of Pretoria Ethical Committee (E087-12).

### SNP quality assessment

SNP quality control and sample pruning was performed by means of Plink (version 1.9) [57]. SNPs with a minor allele frequency of greater than 0.02 and/or genotype rate of less than 0.95 were filtered from the dataset.

### Determination of population structure

One of the SNPs was removed for each pair of SNPs demonstrating an LD of greater than 0.1 in a sliding window of 30 SNPs. Relationship-based pruning was performed and one member of each pair of animals with an observed genomic relatedness of greater than 0.25 was removed from further analyses to correct for population stratification [58]. ADMIXTURE analyses software [59] were subsequently used to determine population structure of unrelated animals. ADMIXTURE was run from K = 2 to K = 10 and a cross-validation procedure was used to ascertain the best k. That k-value that generated the lowest cross-validation standard error was determined as being the most probable population sub-structuring. Q estimate matrices barplots were generated with R [http://cran.r-project.org] for each value of k, and animals were sorted according to ecotypes based on this population structure.

A discriminant analysis of principle components (DAPC) was performed using adegenet 2.0.0 in R [60]. In the absence of group priors, DAPC infers genetic clusters from sequential K-means and model selections. The find.clusters script was utilized to determine clusters with a maximum of 9 groups. The cumulative variance against the number of retained principle components (PCs) (Fig. 4), demonstrated the greatest amount of variance being explained by 100 PCs which were therefore utilized in conjunction with 2 discriminant functions (Fig. 4) to determine group clustering. A scatterplot of the DPCA was subsequently generated.

### Analysis of HPB

PLINK software (http://pngu.mgh.harvard.edu/purcell/plink, [57]) was utilized to impute haplotypes based on single SNP tests for each of the 29 bovine autosomes of 492 Nguni animals. Variants were pruned for LD using an independent pairwise parameters of window size 30, step size 5 and a r^2^ threshold of 0.1. Haplotype blocks were estimated using Haploviews interpretation of Gabriel et al. [61] for each of the 29 bovine autosomes under PLINK’s default block settings. Gene ontology analyses of HPB regions was performed against the *Bos Taurus* reference gene list by means of the PANTHER databases [62].

### Generation of CNV calls and CNV filtering

The Log R ratio, B allele frequency, G type, chromosome and position were exported from GenomeStudio for each animal for analyses using PennCNV [12]. PennCNV has outperformed a number of CNV detection packages on multiple occasions demonstrating a greater specificity , sensitivity for CNV calling and reasonably little bias [26, 63]. PennCNV utilizes a first order Hidden Markov Model, which assumes that the hidden copy number state at each SNP is subject to the copy number state of the most preceding SNP for high resolution CNV discovery with whole genome SNP genotyping data [12]. The Viterbi algorithm is subsequently utilized to determine the most probable sequence of hidden states chromosome by chromosome [12]. A dynamic programming algorithm, the Viterbi algorithm was applied to predict the Viterbi path which generates the most probable sequence of hidden states representing discrete copy numbers along the chromosomes [64].

The PennCNV compile_pfb script [12] was utilized to create a pfb file from the data. The detect_cnv.pl was run to detect CNVs on 29 autosomes. A number of animals (125) exhibited an absolute genomic waviness factor of greater than 0.04. GC content within 1 Mb region (500 K per side) surrounding each marker was calculated and utilized to create the bovine gcmodel. A second analyses including the –gcmodel option was also run for comparative purposes.

In order to minimize the rate of false positives, extensive quality control was applied by means of the filter_cnv.pl script [12]. Two separate filtering criterions were utilized. By means of Golden Helix SVS software, the median DLRS and GCWF values, were utilized to determine the upper outlier threshold set at 1.5 inter-quartile range (IQRs) from the third quartile of all DLRS and GCWF values respectively. Upper outlier thresholds of 0.318 and 0.072 for DLRS and GCWF were thus determined. The second filtering was also performed utilizing more stringent standards where only those CNVs that demonstrated a standard deviation (SD) less than 0.3, B allele drift of less than 0.01 and waviness factor [65] of less than 0.04 were kept.

### Statistical analyses

Bioinformatic tools together with Microsoft Excel software were utilized to organize and analyse the data. A python script developed in house merged overlapping and adjacent CNVs to form CNVRs. Pivot tables summarized data statistics.

### Gene ontology analyeses

RefGene and RefLink annotations (USCS, downloaded on http://genome.ucsc.edu/goldenpath/gbdDescriptionsOld.html) were used to identify genes located within a 10 Mb window surrounding a CNV. Norris & Whan [66] have shown that CNVs have a demonstrated effect on surrounding genes in a number of species. Overlapping CNVs were aggregated to delineate a set of copy number variation regions (CNVRs) [27]. The coincidence of CNVs and corresponding genes identified by the different models was visualized by means of the Pangloss Venn diagram generator (VENNY [67], http://www.pangloss.com/seidel/Protocols/venn4.cgi and GeneVenn http://genevenn.sourceforge.net/vennresults.php). The hypothesis that genes were over or under represented in PANTHER pathways, biological processes, cellular components and molecular functions was tested by means of the bonferoni correction on the pantherdb.org. *Bos taurus* gene ontologies were ascertained by means of the Ensembl and PANTHER databases.

## Additional files

Additional file 1:
**HPBs identified in 492 Nguni cattle and the respective genes they cover or lie in close proximity too.** (XLSX 66 kb) Additional file 2:
**Biological process, molecular function and cellular component representation of those genes covered by HPBs identified in Nguni cattle.** (XLSX 939 kb) Additional file 3:
**Functional classification of genes covered by CNVRs from this study and those of** [5, 29, 30]. (XLSX 1415 kb) Additional file 4:
**CNVRs distribution across individuals and genes covered.** (XLSX 46 kb) Additional file 5:
**a. CNVRs identified in this study and by** [5, 29, 30], **the number of animals in the study (ANMLs), the breed (BRD), the number of genes (GEN), CNVRs identified by other studies (CNVR SHRD) and number of genes that are unique to the study (UNIQ GEN).** b. Comparisons of genes within CNVRs (GEN) identified within this study with those identified within other breeds [5, 29, 30] revealed 402 genes that were unique to the Nguni. (XLSX 17 kb) Additional file 6:
**Tables demonstrating the representation of biological process, molecular function and cellular components by those genes within and or 10 Mb downstream of CNVRs identified in Nguni cattle.** (XLSX 794 kb) Additional file 7:
**HPB CNVR overlap regions, the number of animals presenting the CNVR and the genes covered in Nguni cattle.** (XLSX 33 kb)

## Abbreviations

CNV: Copy number variations
CNVRs: Copy number variation regions
GO: Gene ontology
SNP: Single nucleotide polymorphism
Mb: Megabase
Kb: Kilobase
DLRS: Derivative log ratio spread
GCWF: GC wave factor
SD: Standard deviation
